# Endoscopic vs. Microscopic Transsphenoidal Surgery for the Treatment of Pituitary Adenoma: A Meta-Analysis

**DOI:** 10.3389/fsurg.2021.806855

**Published:** 2022-02-02

**Authors:** Jia Chen, Hongyan Liu, Siliang Man, Geng Liu, Quan Li, Qingyao Zuo, Lili Huo, Wei Li, Wei Deng

**Affiliations:** ^1^Department of Endocrinology, Beijing Jishuitan Hospital, Beijing, China; ^2^Department of Endocrinology, Chinese People's Liberation Army (PLA) General Hospital, Beijing, China; ^3^Department of Rheumatology, Beijing Jishuitan Hospital, Beijing, China; ^4^Department of Emergency, Beijing Jishuitan Hospital, Beijing, China

**Keywords:** pituitary adenoma, endoscopic, meta-analysis, microscopic, transsphenoidal surgery

## Abstract

**Purpose:**

Currently, endoscopic transsphenoidal surgery (ETS) and microscopic transsphenoidal surgery (MTS) are commonly applied treatments for patients with pituitary adenomas. This meta-analysis was conducted to evaluate the efficacy and safety of ETS and MTS for these patients.

**Methods:**

A computer search of Pubmed, Embase, Cochrane library, Web of Science, and Google Scholar databases was conducted for studies investigating ETS and MTS for patients with pituitary adenomas. The deadline is March 01, 2021. RevMan5.1 software was used to complete this meta-analysis after literature screening, data extraction, and literature quality evaluation.

**Results:**

A total of 37 studies including 5,591 patients were included. There was no significant difference in gross tumor removal (GTR) and hormone-excess secretion remission (HES remission) between two groups [RR = 1.10, 95% CI (0.99–1.22), *P* = 0.07; RR = 1.09, 95% CI (1.00–1.20), *P* = 0.05]. ETS was associated with lower incidence of diabetes insipidus (DI) [RR = 0.71, 95% CI (0.58–0.87), *P* = 0.0008], hypothyroidism [RR = 0.64, 95% CI (0.47–0.89), *P* = 0.007], and septal perforation [RR = 0.32, 95% CI (0.13–0.79), *P* = 0.01] than those with MTS.

**Conclusion:**

This meta-analysis indicated that ETS cannot significantly improve GTR and HES remission. However, ETS could reduce the incidence of DI, hypothyroidism, and septal perforation without increasing the rate of other complications.

**Systematic Review Registration:**

https://www.crd.york.ac.uk/prospero/#myprospero, identifier: CRD42021241217.

## Introduction

Rapid progressive pituitary adenomas is one of the most common intracranial tumors, constituting nearly 10% of intracranial tumors (1). It often leads to abnormal secretion of pituitary hormones, abnormal vision, and other nerve and brain damages, and ultimately endanger the life of patients. Medication, radiotherapy, and surgery are the main treatments. In addition to a prolactinoma, usually treated with dopamine agonists, surgery was considered the most effective method. However, due to the pivotal location of pituitary adenomas in the pituitary fossa and critical structures such as the hypothalamus, cavernous sinus, and internal carotid artery nearby, traditional surgery is difficult to remove tumors completely. In 1907, Schloffer first proposed transsphenoidal removal of pituitary adenomas (2). Since 1960, microscopic transsphenoidal surgery (MTS) is the preferred choice for pituitary adenoma because of its high cure rate and low incidence of complication. However, there are still some limitations, especially the poor vision above and to the side of the saddle area.

With the development of endoscopic technology, surgery has been developed rapidly. In 1992, Jankowski adopted endoscopic technology in the procedure of pituitary adenomas (3). In 2007, Laufer et al. found that endoscopic transsphenoidal surgery (ETS) is safer (4).

In recent years, some newly published controlled trials investigated the efficacy and safety between ETS and MTS for the treatment of pituitary adenomas. However, the results are inconclusive. This meta-analysis was conducted based on these inconclusive results.

## Methods

### Protocol and Registration

This meta-analysis was conducted based on the Preferred Reporting Items for Systematic reviews and meta-analyses (PRISMA) recommendations. Previously, we complete a protocol registration on PROSPERO with the number CRD42021241217.

### Search Strategy

A search of Pubmed, Embase, Cochrane Controlled Center Register of Controlled Trials (CENTRAL), and Web of Science was performed (cutoff was set on March 01, 2021). Keywords include “microscopic,” “endoscopic,” “Cushing Syndrome,” “Cushing's Disease,” “pituitary adenoma,” “Pituitary Adenomas,” “Acromegaly,” “transsphenoidal.” References of included studies were screened for additional potential trials. No language restriction was applied. Endnote X9 (Thomson Reuters, New York, USA) was used to remove duplicate documents. Titles, abstracts, and full texts were screened respectively to select studies that met the inclusion criteria. We also supplemented it with Google Scholar and further searched the references of the selected articles.

### Inclusion and Exclusion Criteria

#### Inclusion Criteria

(1) Research types: prospective cohort study (PCS), retrospective cohort study (RCS) or case-control study publicly published at home and abroad; (2) Research objects: patients with pituitary adenomas aged >18 years; (3) Intervention measures: experimental group using ETS and the control group was treated with MTS; (4) Main outcomes were: gross tumor removal (GTR), hormone-excess secretion remission (HES remission), treatment-related adverse events (TARE) including cerebrospinal fluid (CSF) leak, diabetes insipidus (DI), epistaxis, hypopituitarism, meningitis, overall complication, visual improvement, Visual loss, hyponatremia/(SIADH), and septal perforation.

#### Exclusion Criteria

Articles that do not meet the inclusion criteria, cannot obtain the main indicators in the article, and have not received a response through contacting the author and republished articles were excluded.

### Data Extraction

General characteristics of the included studies were obtained through reading the full text, as well as the inclusion criteria, interventions, follow-up, main outcomes, etc. For unavailable data, we would try to contact the author through emails. Data extraction was performed by two authors independently. If there is any inconsistency or disagreement in the data, first discuss it. If it still cannot be resolved, the third author will resolve it.

### Literature Quality Evaluation

Two authors respectively assessed the included studies according to The Newcastle–Ottawa Scale, NOS. The quality evaluation contains three aspects with eight items: Selection, Comparability, and Exposure.

### Statistical Analysis

RevMan5.1 software was used to complete this meta-analysis. The chi-square test and *I*^2^ test were used to analyze the heterogeneity among the studies. When low homogeneity among studies was observed (*I*^2^ <50%, *p* > 0.1), the fixed effects model would be adopted; otherwise, the random-effects model would be employed. The descriptive analysis would be performed once clinical data cannot be meta-analyzed.

### Patient and Public Involvement

The design or conduct of this meta-analysis did not involve the patient or the public.

## Results

### Literature Screening and Characteristics of Included Studies

**Figure 1:**
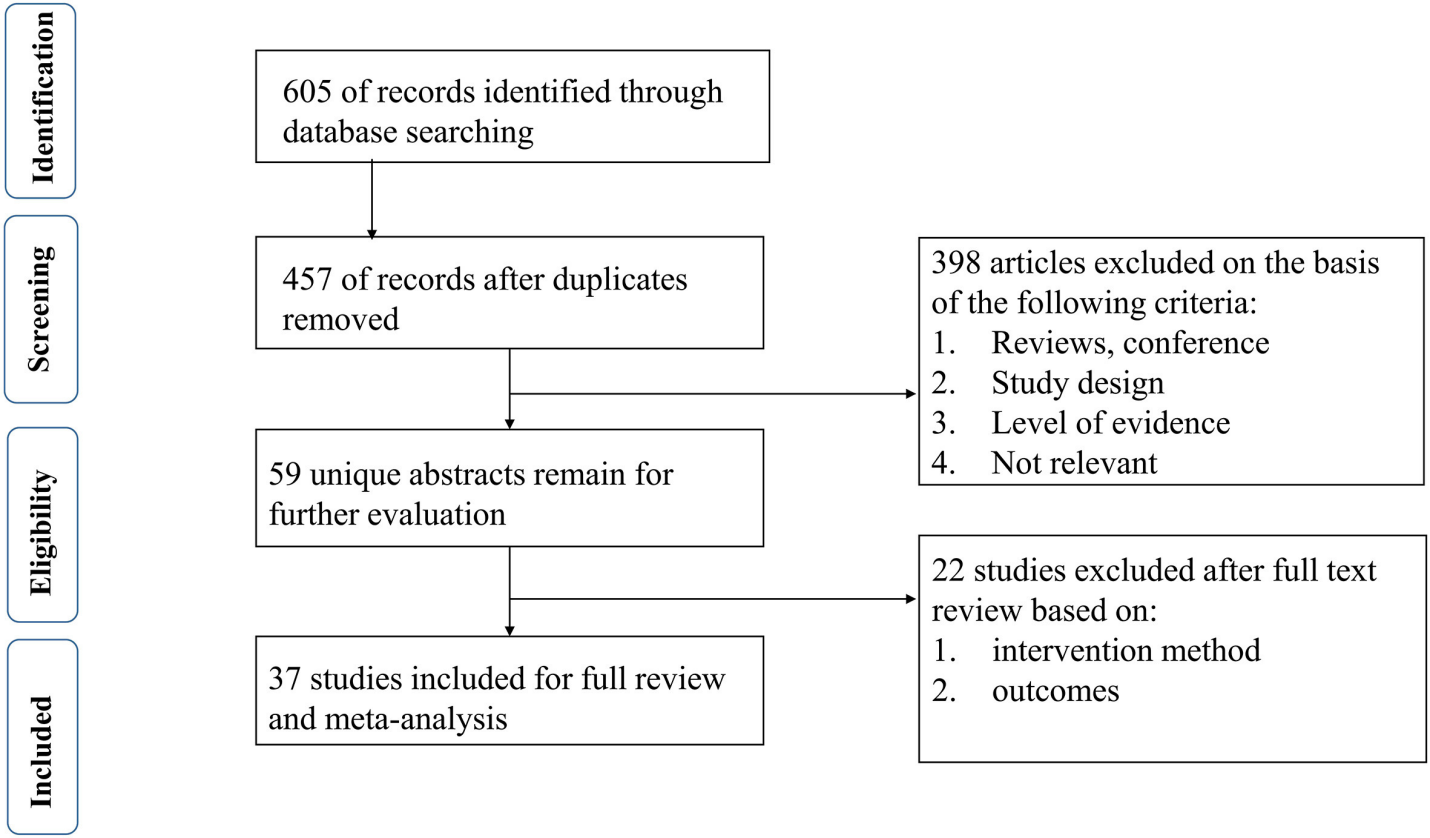
Flow diagram showing the study selection process.

Six hundred and five studies containing 601 initially retrieved and 4 obtained through screening reference list of the included studies were identified. One hundred forty-eight duplicate articles were eliminated by EndNote software. Three hundred ninety-eight studies were abandoned after checking the title and abstract. Twenty-two studies were removed based on the full-text screen, and finally, 37 (5–41) studies with 5,591 participants were confirmed to meet the inclusion criteria. Patients mainly come from Europe, North America, and South America. More details are summarized in Table 1. The literature selection process is shown in Figure 1.

**Table 1:** Characteristics of included studies.

**References**	**Study period**	**Study design**	**Sample size (ETS/MTS)**	**Country**	**Disease**	**Age**		**Sex**	
						**ETS**	**MTS**	**ETS**	**MTS**
Bora et al. (40)	2009.1–2009.6	R	55/47	India	CD	28 (9–55)		
Castaño-Leon et al. (41)	1995–2017	P	97/90	Spain	CD	52 (IQR = 22)	45 (IQR = 24)	39/58	36/54
Broersen et al. (42)	1978–2016	P	50/87	Netherlands	CD	44.4 (15.1)	38.9 (15.4)	16/34	18/69
Little et al. (38)	2006.1–2014.12	P	177/82	Argentina	nFPAs	58.1 (14.0)	58.6 (13.3)	104/73	52/30
Pablo et al. (39)	2011.3–2014.12	R	140/259	Argentina	PAs	48.5 (18–85)	51 (17–90)	61/79	109/150
Agam et al. (32)	1992.11–2017.3	R	170/983	USA	PAs	53.3 (13.6)	49.1 (16.6)		
Akbari et al. (34)	2012–2014	R	16/19	Iran	PAs	39.43 (15.2)	43.06 (11.29)	19/16
Prajapati et al. (35)		P	17/11	India	PAs	41.1 (11.8)	41.9 (13.2)	19/11
Wang et al. (36)	2003.1–2012.8	R	117/37	USA	PAs	50	52	54/63	10/27
Eseonu et al. (31)	2005.5–2005.8	R	275/109	USA	PAs	49.0 ± 16.2	48.8 ± 15.8	163/221
Levi et al. (32)	2004–2012	R	140/81	Italy	PAs	58.5	52	130/91
Zaidi et al. (30)	2011.10–2016.6	P	55/80	USA	PAs	55.9 (13.8)	59.1 (14.6)	85/50
Fathalla et al. (36)	2000–2013	R	42/23	Canada	Acromegaly	43.2	42.1	21/21	7/16
Karppinen et al. (27)		R	41/144	Finland	nFPAs	58.5 ± 16	58.4 ± 13	118/67
Lenzi et al. (28)	1996–2006	R	22/15	Italy	Acromegaly				
Little et al. (29)	2011.10–2013.8	P	107/111	USA	PAs	52.1 (15.4)	51.8 (17.1)	104/114
Dallapiazza et al. (23)	2010.6–2013.1	R	56/43	USA	nFPAs	56.2 ± 12.8	56.7 ± 16.9	51/48
Halvorsen et al. (24)	2020.9–2011.2	R	238/268	Norway	PAs			291/215
Sarkar et al. (25)	2005.1–2013.4	R	66/47	India	Acromegaly	37.6 ± 10.8	38.7 ± 12.2	30/36	26/21
Alahmadi et al. (19)	2000–2010	R	17/25	Canada	CD			11/31
Kahilogullari et al. (20)	2010–2012	P	25/25	Austria	PAs	40.84 (12.56)	46.56 (7.75)	4/21	6/19
Razak et al. (21)	2008.1	R	40/40	UK	PAs	47.4	49.3	19/21	22/18
Starke et al. (22)	2004.8–2009.10	R	72/41	USA	acromegaly	49.2 (14.9)	47.5 (14.2)	40/32	20/21
Cheng et al. (16)	2003.7–2009.7	R	68/59	China	FPAs	37.2	33.8	51/76
Massimi et al. (17)	2000–2005	R	17/14	Italy	PAs	10.2	11.4	14/17
Messerer et al. (18)	2006–2009	R	82/82	France	nPAs	57	56.9	98/66
D'Haens et al. (15)	1995.2–2007.1	R	60/60	Belgium	FPAs				
Choe et al. (12)	1997–2004	R	12/11	Korea	FPAs	47 ± 12	48 ± 10	7/5	9/2
Higgins et al. (13)		R	19/29	UK	PAs	54.2	52.8	25/23
O'Malley et al. (14)	2003.7–2008.5	R	25/25	USA	PAs	47.9 (18–73)	50.8 (23–78)	15/10	16/9
Jain et al. (10)		P	10/10	India	PAs	40.1	31.9	9/11
Neal et al. (11)	1999–2004	R	21/15	USA	PAs	51	39	9/12	5/10
Casler et al. (9)	1996.11–2003.7	R	15/15	USA	PAs	41.6	50.66	6/9	10/5
White et al. (8)	1996–2002	R	50/50	USA	PAs	41.1	43.5	24/26	33/17
Cappabianca et al. (5)	1997.1–1997.6	R	10/20	USA	PAs	33–67	20–68	6/4	11/9
Koren et al. (6)	1993.1–1997.6	R	20/20	Israel	PAs				
Sheehan et al. (7)	1995.1–1997.10	R	26/44	USA	nFPAs	59.2 (15.1)	57.8 (14.9)	18/8	31/13

*R, Retrospective; P, Prospective; PAs, pituitary adenomas; CD, Cushing Disease; FPAs, functioning Pituitary Adenomas; nFPAs, nonfunctioning Pituitary Adenomas; ETS, endoscopic transsphenoidal surgery; MTS, microscopic transsphenoidal surgery*.

### Methodological Quality

Tables 2, 3 showed the details of the quality evaluation of all included studies.

**Table 2:** Assessment of quality of included retrospective studies.

**References**	**Selection**				**Comparability**		**Exposure**		
	**Is the case definition adequate**	**Representativeness of the cases**	**Selection of controls**	**Definition of controls**	**Comparability of cases and controls: most important factor**	**Comparability of cases and controls: a second important factor**	**Ascertainment of exposure**	**Same method of ascertainment for cases and controls**	**Non-response rate**
Bora et al. (40)	Y	Y	Y	Y	Y	N	Y	Y	Y
Pablo et al. (39)	Y	Y	Y	Y	Y	N	Y	Y	Y
Agam et al. (33)	Y	Y	Y	Y	Y	N	Y	Y	Y
Akbari et al. (34)	Y	Y	Y	Y	Y	N	Y	Y	Y
Wang et al. (36)	Y	Y	Y	Y	Y	N	Y	Y	Y
Eseonu et al. (31)	Y	Y	Y	Y	Y	N	Y	Y	Y
Levi et al. (32)	Y	Y	Y	Y	Y	N	Y	Y	Y
Fathalla et al. (26)	Y	Y	Y	Y	Y	N	Y	Y	Y
Karppinen et al. (27)	Y	Y	Y	Y	Y	N	Y	Y	Y
Lenzi et al. (28)	Y	Y	Y	Y	Y	N	Y	Y	Y
Dallapiazza et al. (23)	Y	Y	Y	Y	Y	N	N	Y	Y
Halvorsen et al. (24)	Y	Y	Y	Y	Y	N	Y	Y	Y
Sarkar et al. (25)	Y	Y	Y	Y	Y	N	N	Y	Y
Alahmadi et al. (19)	Y	Y	Y	Y	Y	N	Y	Y	Y
Razak et al. (21)	Y	N	Y	Y	Y	N	N	Y	Y
Starke et al. (22)	Y	Y	Y	Y	Y	N	N	Y	Y
Cheng et al. (16)	Y	Y	Y	Y	Y	N	Y	Y	Y
Massimi et al. (17)	Y	N	Y	Y	Y	N	Y	Y	Y
Messerer et al. (18)	Y	N	Y	Y	Y	N	Y	Y	Y
D'Haens et al. (15)	Y	Y	Y	Y	Y	N	Y	Y	Y
Choe et al. (12)	Y	N	Y	Y	Y	N	Y	Y	Y
Higgins et al. (13)	Y	Y	Y	Y	Y	N	Y	Y	Y
O'Malley et al. (14)	Y	Y	Y	Y	Y	N	Y	Y	Y
Neal et al. (11)	Y	Y	Y	Y	Y	N	Y	Y	Y
Casler et al. (9)	Y	N	Y	Y	Y	N	Y	Y	Y
White et al. (8)	Y	N	Y	Y	Y	N	Y	Y	Y
Cappabianca et al. (5)	Y	N	Y	Y	Y	N	Y	Y	Y
Koren et al. (6)	Y	N	Y	Y	Y	N	Y	Y	Y
Sheehan et al. (7)	Y	N	Y	Y	Y	N	Y	Y	Y

*Y, Yes; N, No*.

**Table 3:** Assessment of quality of included prospective studies.

**References**	**Selection**				**Comparability**		**outcome**		
	**Representativeness of treated arm**	**Selection of the comparative treatment arm(s)**	**Ascertainment of the treatment regimen**	**Demonstration that outcome of interest was not present at start of study**	**Comparability between patients in different treatment arms: main factor**	**Comparability between patients in different treatment arms: secondary factor**	**Assessment of outcome with independency**	**Adequacy of follow-up length (to assess outcome)**	**Lost to follow-up acceptable (<10% and reported)**
Castaño-Leon et al. (41)	Y	Y	Y	Y	Y	N	N		Y
Broersen et al. (37)	Y	Y	Y	Y	Y	N	N		Y
Little et al. (38)	Y	Y	Y	Y	Y	N	N		Y
Prajapati et al. (35)	Y	Y	Y	Y	Y	N	N		Y
Zaidi et al. (30)	Y	Y	Y	Y	Y	N	N		Y
Little et al. (29)	Y	Y	Y	Y	Y	N	N		Y
Kahilogullari et al. (20)	Y	Y	Y	Y	Y	N	N		Y
Jain et al. (10)	Y	Y	Y	Y	Y	N	N		Y

*Y, Yes; N, No*.

### Outcomes

#### GTR

**Figure 2:**
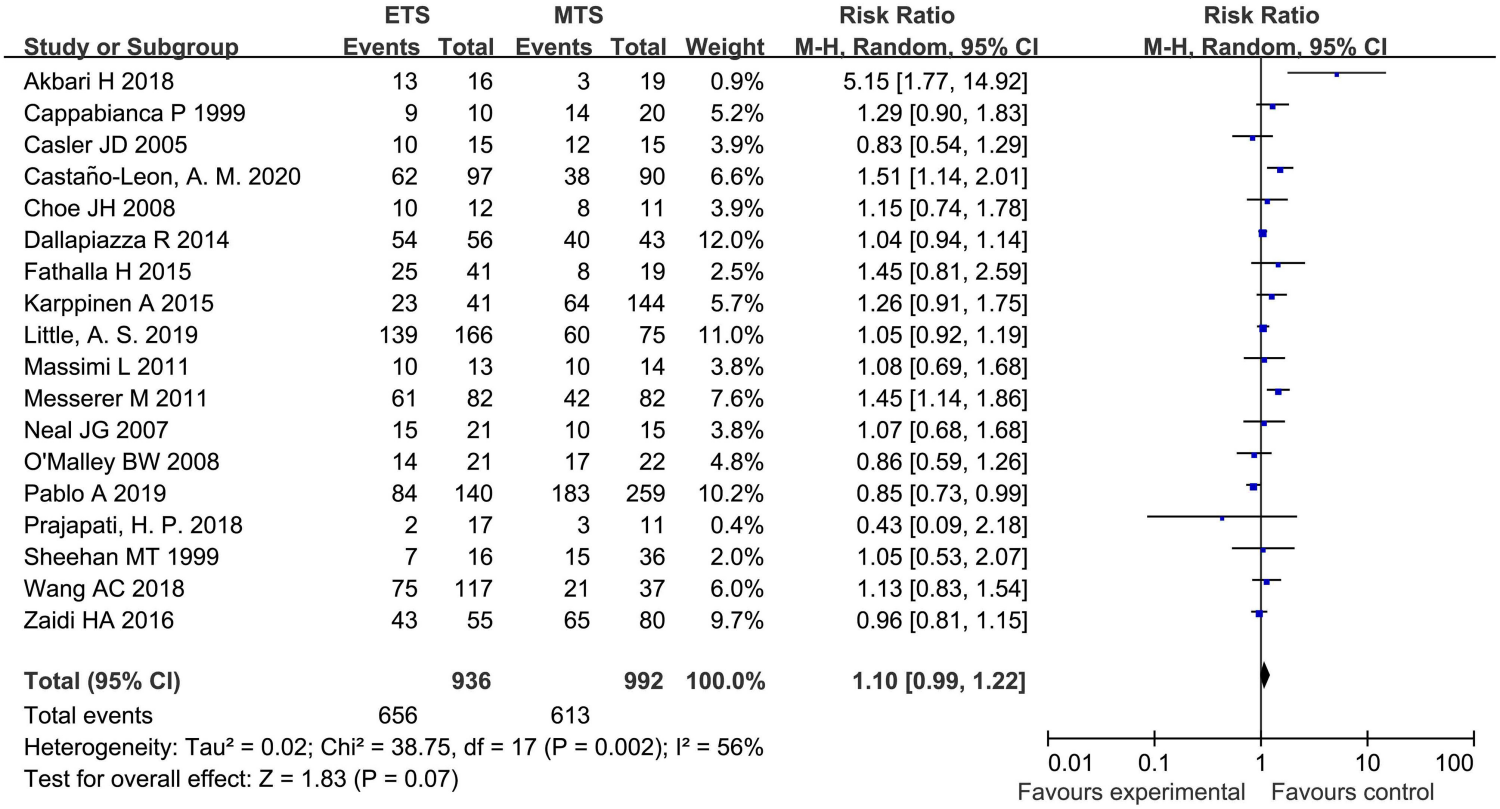
Effect of ETS on GTR, compared with MTS.

Eighteen studies analyzed the GTR between ETS and MTS for pituitary adenoma. No significant difference was observed in GTR between two groups [RR = 1.10, 95% CI (0.99–1.22), *P* = 0.07; Figure 2].

#### HES Remission

**Figure 3:**
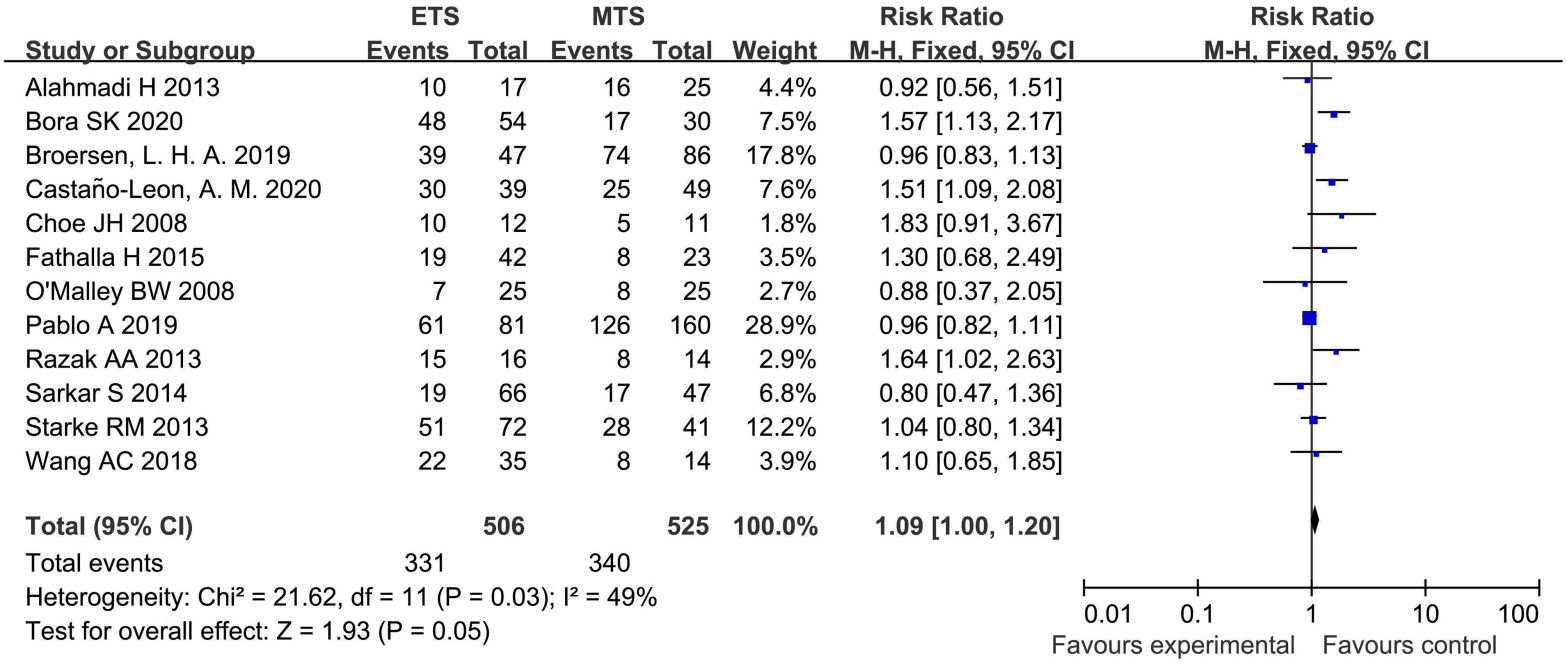
Effect of ETS on HES remission, compared with MTS.

Twelve studies analyzed the HES remission between ETS and MTS for pituitary adenoma. Results indicated that there was no significant difference in HES remission between two groups [RR = 1.09, 95% CI: (1.00–1.20), *P* = 0.05; Figure 3].

#### Overall Complication

**Figure 4:**
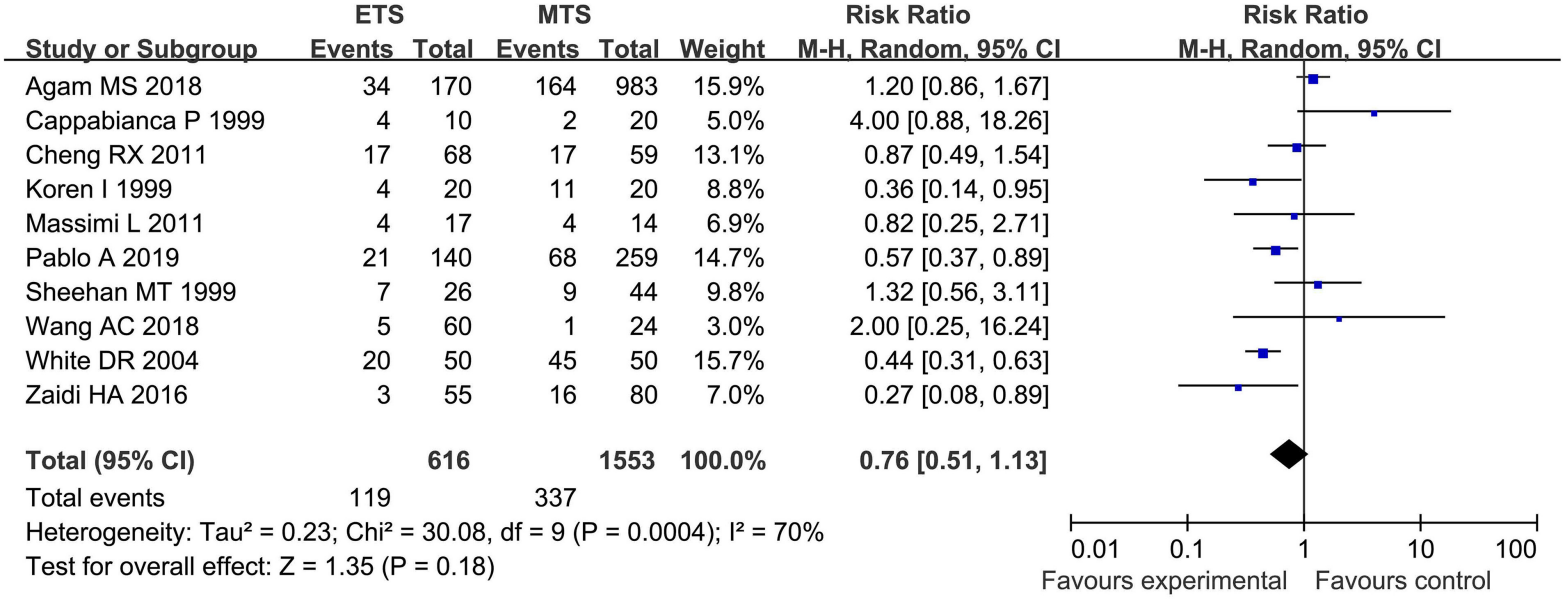
Effect of ETS on overall complication, compared with MTS.

Ten studies investigated the overall complication between ETS and MTS for pituitary adenoma. Results demonstrated that there was no significant difference in overall complication between ETS group and MTS group [RR = 0.76, 95% CI: (0.51–1.33), *P* = 0.18, Figure 4].

#### Cerebrospinal Fluid Leak

**Figure 5:**
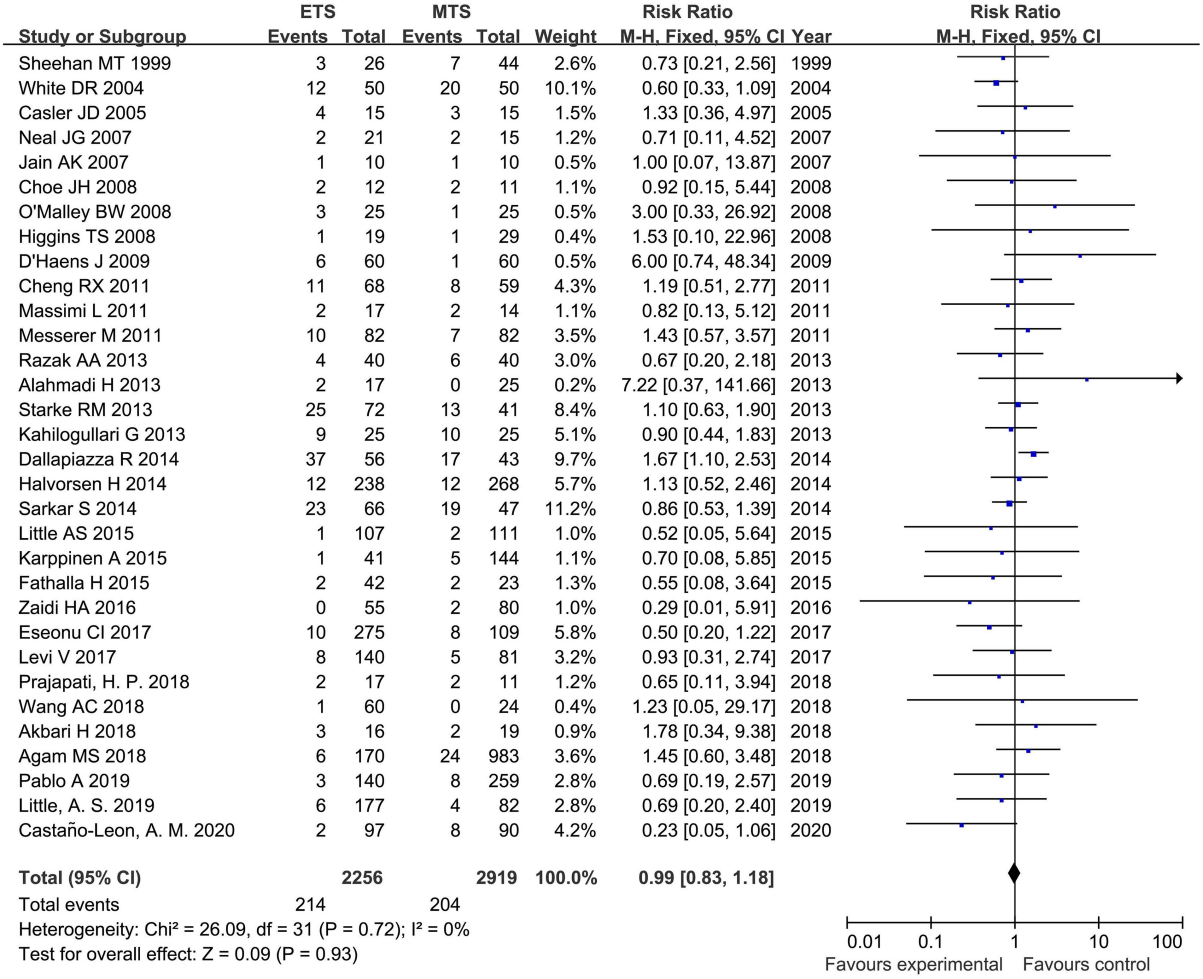
Effect of ETS on cerebrospinal fluid (CSF), leak MTS.

Thirty-two studies analyzed the CSF leak of ETS and MTS for pituitary adenoma. There was no significant difference was observed regarding on incidence of CSF leak between ETS group and MTS group [RR = 0.99, 95% CI: (0.83–1.18), *P* = 0.05] (Figure 5).

#### Diabetes Insipidus

**Figure 6:**
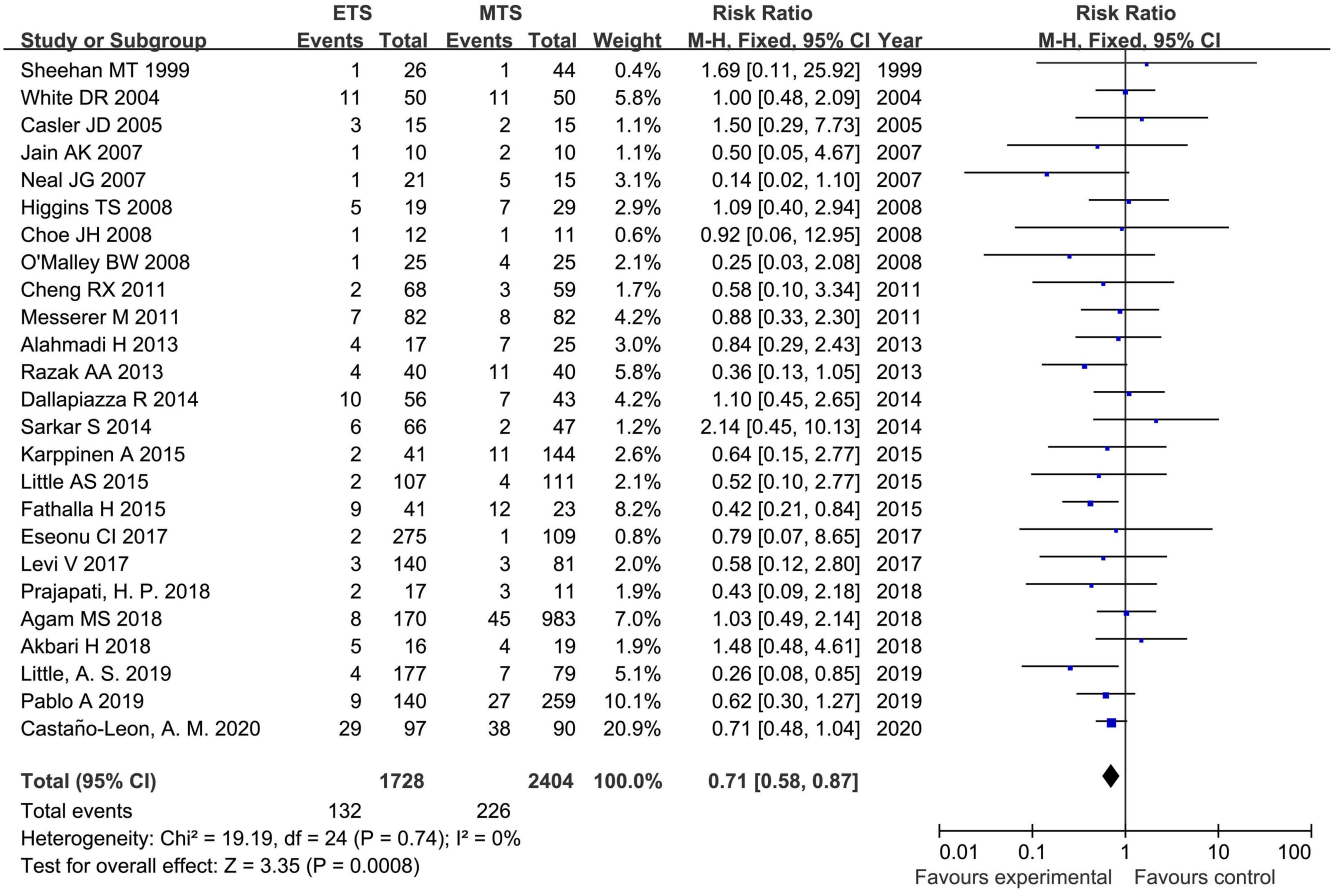
Effect of ETS on diabetes insipidus (DI), compared with MTS.

Twenty-five studies analyzed the DI of ETS and MTS for pituitary adenoma. For DI, ETS group related to a significantly lower rate in comparison to MTS group [RR = 0.71, 95% CI: (0.58–0.87), *P* = 0.0008, Figure 6).

#### Syndrome of Inappropriate Antidiuretic Hormone Secretion (SIAHD)

**Figure 7:**
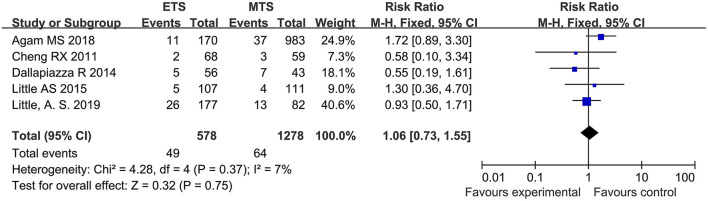
Effect of ETS on SIAHD, compared with MTS.

Five studies analyzed the SIAHD of ETS and MTS for pituitary adenoma. No significant difference was observed in SIAHD between two groups [RR = 106, 95% CI: (0.73–1.55), *P* = 0.75; Figure 7].

#### Septal Perforation

**Figure 8:**
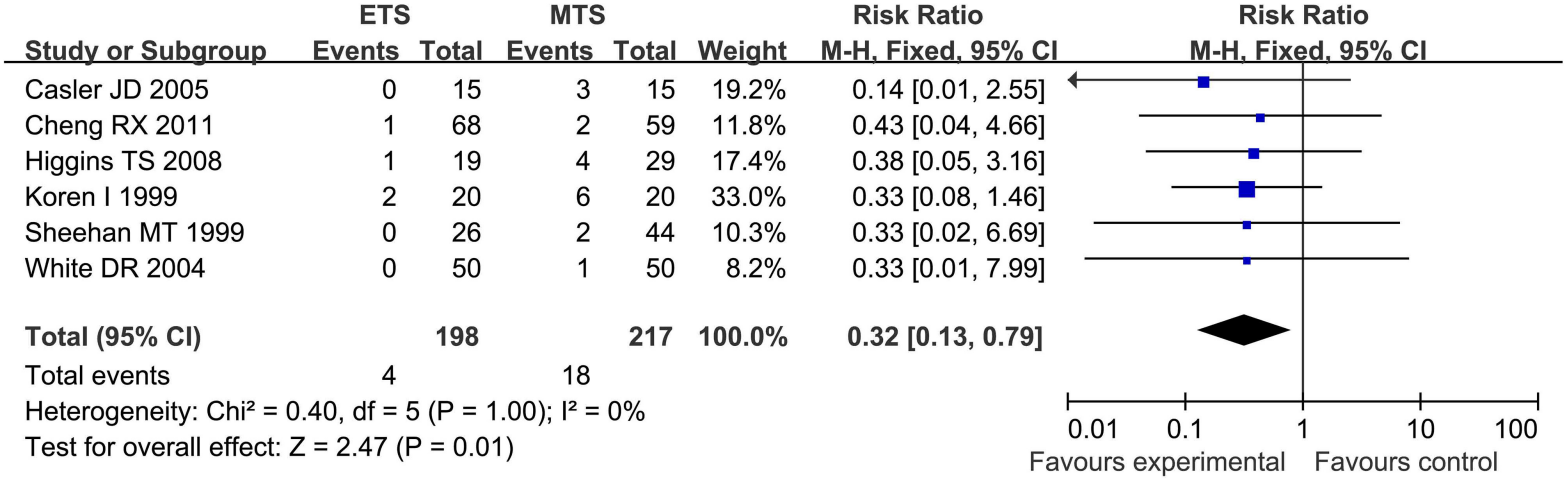
Effect of ETS on septal perforation, compared with MTS.

Six studies analyzed the septal perforation of ETS and MTS for pituitary adenoma. ETS group showed a significantly lower rate in comparison to MTS group [RR = 0.32, 95% CI: (0.13–0.79), *P* = 0.01; Figure 8].

#### Hypothyroidism

**Figure 9:**
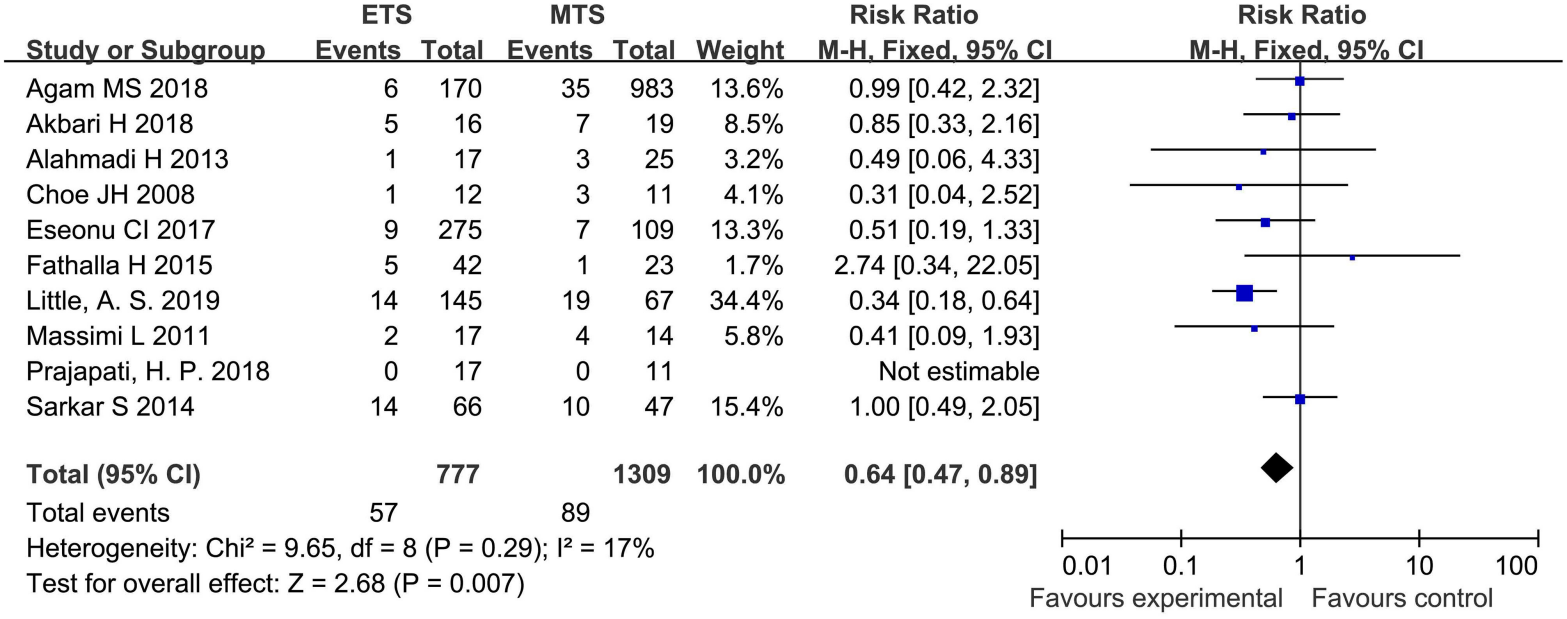
Effect of ETS on hypothyroidism, compared with MTS.

Ten studies analyzed the hypothyroidism of ETS and MTS for the treatment of pituitary adenoma. The results showed that the hypothyroidism in the ETS group was significantly lower than that in the MTS group [RR = 0.64, 95% CI: (0.47–0.89), *P* = 0.007; Figure 9].

#### Visual Improvement

**Figure 10:**
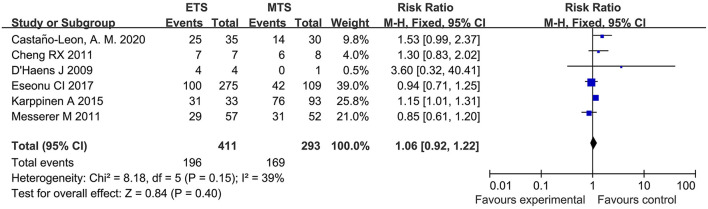
Effect of ETS on visual improvement, compared with MTS.

Six studies investigated the visual improvement of ETS and MTS for the treatment of pituitary adenoma. No significant difference was observed in visual improvement between two groups [RR = 1.06, 95% CI: (0.92–1.22), *P* = 0.40, Figure 10].

#### Visual Loss

**Figure 11:**
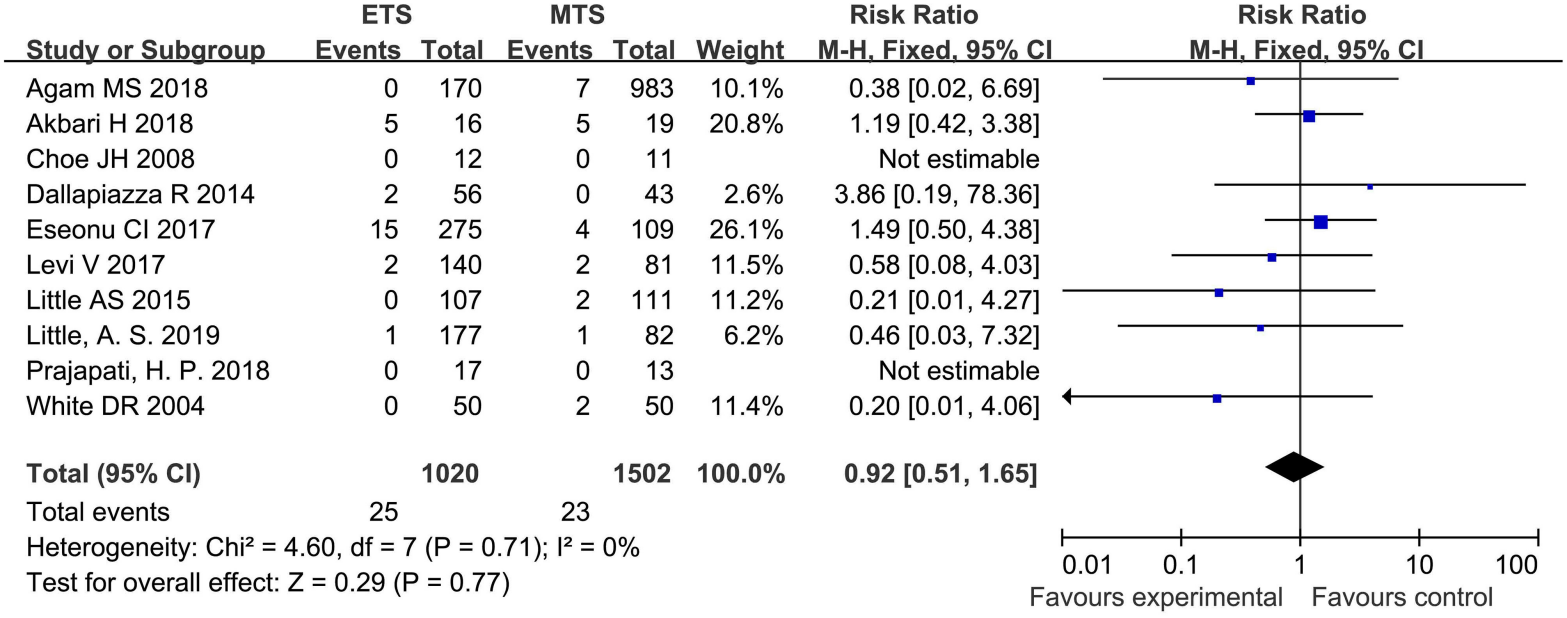
Effect of ETS on visual loss, compared with MTS.

Ten studies analyzed the visual loss of ETS and MTS for the treatment of pituitary adenoma. No significant difference was observed in visual loss between two groups [RR = 0.92, 95% CI: (0.51–1.65), *P* = 0.77; Figure 11].

#### Epistaxis

**Figure 12:**
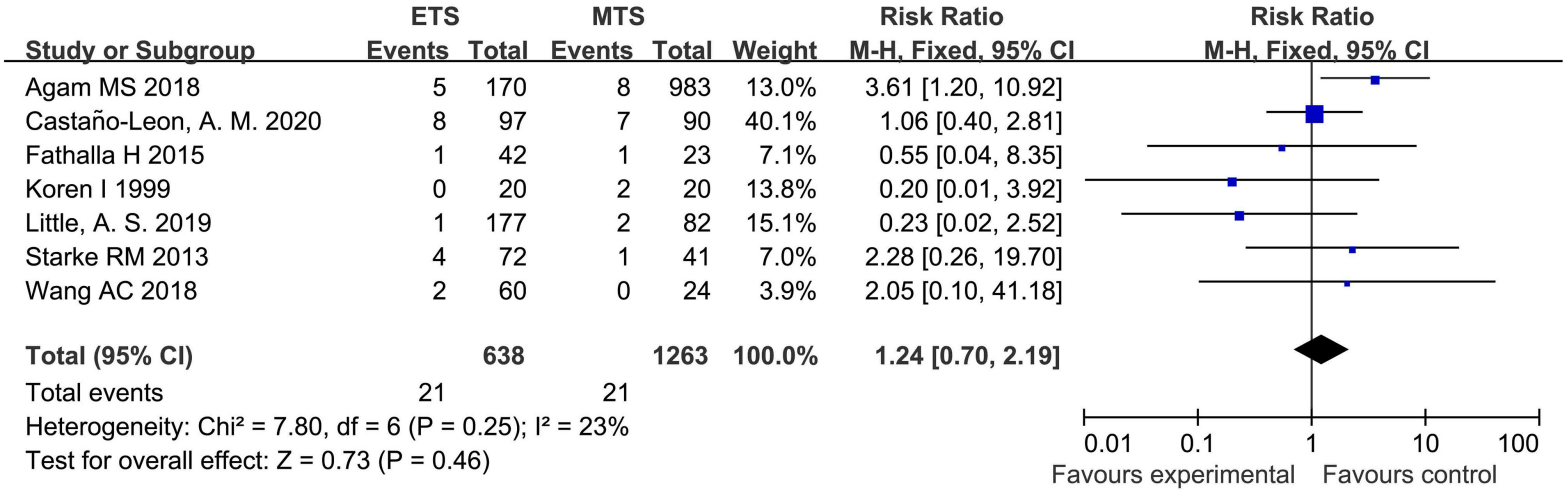
Effect of ETS on epistaxis, compared with MTS.

Seven studies analyzed the epistaxis of ETS and MTS for the treatment of pituitary adenoma. Results indicated that no significant difference was observed in epistaxis between two groups [RR = 1.24, 95% CI: (0.70–2.19), *P* = 0.46; Figure 12].

#### Meningitis

**Figure 13:**
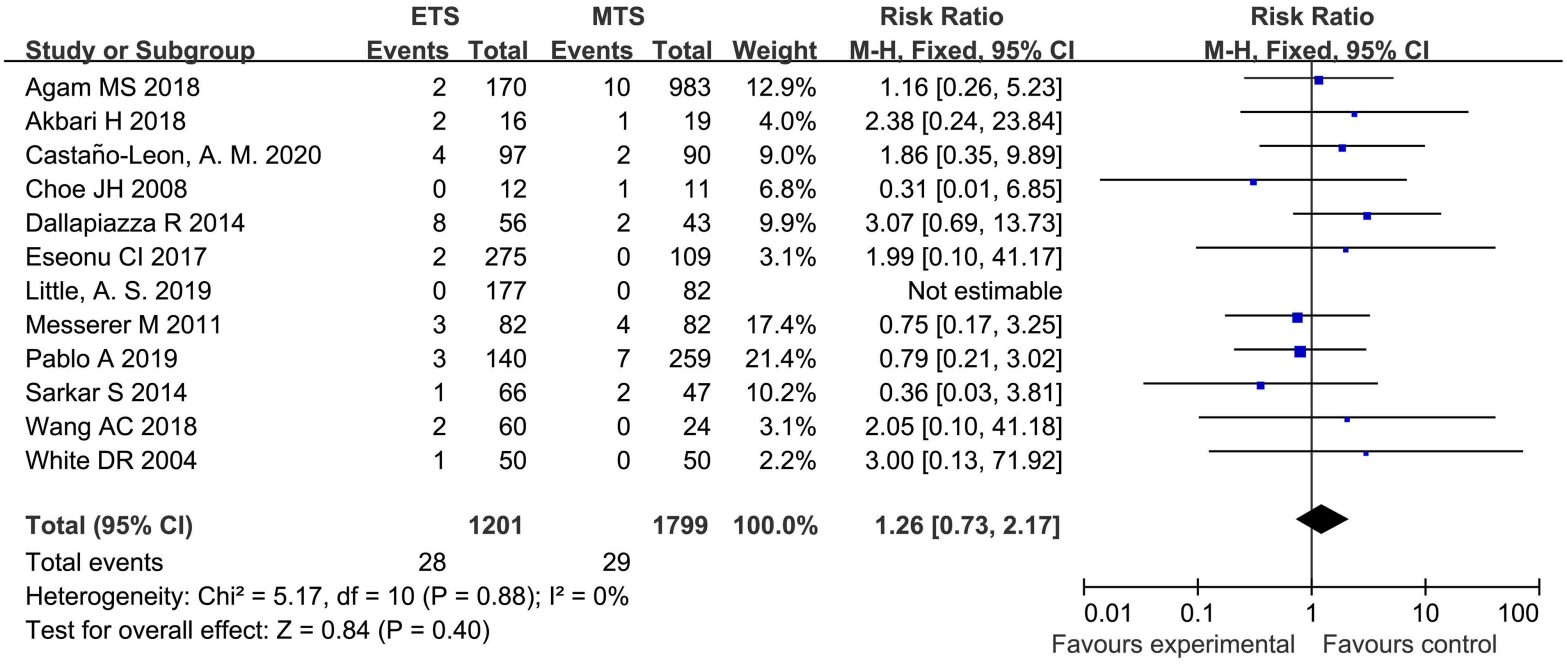
Effect of ETS on meningitis, compared with MTS.

Twelve studies analyzed the meningitis of ETS and MTS for the treatment of pituitary adenoma. Results indicated that no significant difference was observed in meningitis between two groups [RR = 1.26, 95% CI: (0.73–2.17), *P* = 0.40; Figure 13].

#### Publication Bias

The funnel plot results showed there is no publication bias (Supplementary Materials).

## Discussion

At present, the standard surgical method for pituitary adenomas is ETS or MTS. However, it is still controversy concerning the short-term effects of these two surgical methods.

This meta-analysis included a total of 37 controlled studies to study the efficacy and safety of ETS and MTS for the treatment of pituitary adenomas. Main outcomes included: 1. clinical efficacy: no significant difference was observed concerning GTR, HES remission, and visual improvement between two surgical treatments; 2. clinical safety: ETS could decrease the incidence of diabetes DI, hypothyroidism as well as septal perforation. However, no significant difference was observed regarding CSF leak, epistaxis, meningitis, overall complication, visual loss, and SIADH between the two methods.

Although the two methods do not show significant differences in GTR, ETS has its unique advantages in relatively tricky operations. The application of angled endoscopy, with a large range of movement, could ensure the removal of tumors which cannot be realized through the traditional transsphenoidal approach (4, 42). Secondly, due to the flexibility, the ETS can be inserted into the resected tumor cavity to explore residual tumors, which means that intraoperative MRI would be unnecessary in these cases (43). Besides, for large tumors possibly accompanied by CSF leakage, the panoramic field of the endoscope has its advantage (44–46).

Various factors could influence postoperative vision recovery, such as the age of onset, the degree of preoperative visual field defect, and the size of the tumor. The vision of most patients could be improved after surgery. However, no evidence demonstrated that the selection of surgical methods can affect the recovery of patients' postoperative vision. Our outcomes also indicated a similar conclusion.

For most patients, the postoperative DI is transient. Only a few patients will progress to permanent DI. Besides, the surgical precision has an impact on the occurrence of DI (14, 47). The reduced incidence of DI of ETS may benefit from the fact that endoscopy can ensure relatively good vision during the operation.

CSF leak is considered a common postoperative complication. The lower rate of postoperative CSF leak in patients treated by ETS has been reported (48, 49) which is possibly associated with the following facts: First, endoscopy can detect the lesion tissue and its surrounding structures. Second, the blind corners under microscopy could be observed through different angles of the endoscope. However, our results indicated that no significant difference was observed concerning the rate of postoperative CSF leak between two surgical treatments. Three potential reasons were: 1. the included studies are all observational studies with a relatively low level of evidence-based. 2. The sample size is not large. 3. The rate of CSF leak is not significantly influenced by surgical methods. A large number of high-quality randomized controlled studies were necessary for further confirmation.

Septal perforation is a common postoperative complication for patients treated by MTS due to the use of a retractor during the intraoperative procedure. ETS hardly damages septum nasi because it is unnecessary to adopt a retractor and the approach is the natural passage of the human body (50).

The present analysis has several limitations. First, there were no RCTs in the meta-analysis. Second, most of the included studies did not describe the evaluation method of the GTR in detail. Third, it is impossible to evaluate the relationship of the postoperative results and the classification of pituitary adenomas because no subgroup analysis was conducted according to the classification of pituitary adenomas in major studies included.

## Conclusion

This meta-analysis found that endoscopic transsphenoidal surgery cannot significantly decrease GTR and HES remission, but it could decrease the rate of DI, hypothyroidism, and septal perforation without increasing the rate of other complications.

## Data Availability Statement

The original contributions presented in the study are included in the article/Supplementary Material, further inquiries can be directed to the corresponding author/s.

## Author Contributions

WD designed the research and had primary responsibility for the final content. JC and HL conducted the research. SM, GL, and QL analyzed the data. JC, QZ, and LH wrote the manuscript. WL revised the manuscript. All authors approved the final manuscript.

## Funding

This article was funded by the National Key Research and Development Program of China (No. 2020YFC2004900).

## Conflict of Interest

The authors declare that the research was conducted in the absence of any commercial or financial relationships that could be construed as a potential conflict of interest.

## Publisher's Note

All claims expressed in this article are solely those of the authors and do not necessarily represent those of their affiliated organizations, or those of the publisher, the editors and the reviewers. Any product that may be evaluated in this article, or claim that may be made by its manufacturer, is not guaranteed or endorsed by the publisher.
