# Supplementary material for: Endoscopic vs. Microscopic Transsphenoidal Surgery for the Treatment of Pituitary Adenoma: A Meta-Analysis
Source: Front Surg. 2022 Feb 2;8:806855. doi: 10.3389/fsurg.2021.806855 (PMC8847202; doi:10.3389/fsurg.2021.806855)
Supplement: Supplementary file 1 [file Data_Sheet_1.docx]

**Supplementary Materials**

**Endoscopic vs. Microscopic Trans-Sphenoidal Surgery for the Treatment of Pituitary Adenoma: a meta-analysis**





Figure S1 funnel plot of CSF leak





Figure S2 funnel plot of DI





Figure S3 funnel plot of Epistaxis





Figure S4 funnel plot of GTR





Figure S5 funnel plot of HES remission





Figure S6 funnel plot of Hypopituitarism





Figure S7 meningitis





Figure S8 funnel plot of overall complication





Figure S9 funnel plot of septal perforation





Figure S10 funnel plot of SIADH





Figure S11 funnel plot of Visual improvement





Figure S12 funnel plot of worsened vision
